# Non‑surgical outcomes and risk factors for pulmonary metastasis from giant cell tumor of bone

**DOI:** 10.3892/ol.2023.14095

**Published:** 2023-10-11

**Authors:** Thanate Poosiripinyo, Sermsak Sukpanichyingyong, Krits Salang, Wiriya Mahikul, Thanapon Chobpenthai

**Affiliations:** 1 Department of Orthopedics, Khon Kaen Hospital, Mueang Khon Kaen, Khon Kaen 40000, Thailand; 2 Department of Orthopedics, Princess Srisavangavadhana College of Medicine, Chulabhorn Royal Academy, Bangkok 10210, Thailand

**Keywords:** giant cell tumor, pulmonary metastasis, bone tumor, non-surgical, risk factor

## Abstract

The present study detailed four factors associated with an increased risk of pulmonary metastasis, age, pathological fracture, local recurrence and mode of treatment. Local recurrence and pathological fracture were independent risk factors for developing metastasis. From January 2016 to December 2021, data from 50 patients diagnosed with giant cell tumor of bone (GCTB) treated in Khon Kaen Hospital, Thailand, were retrospectively analyzed. The risk factors, including age at diagnosis, location of GCTB, clinical presentation, Campanacci stage and no. of local recurrences, for GCTB-induced pulmonary metastasis were evaluated using univariate and multivariable logistic regression analyses. Of the 50 patients analyzed, 9 patients (18%), with a mean age of 46.3 years (range, 18–68 years), developed pulmonary metastasis. No patients died from pulmonary metastasis in the present study. Statistically significant associations were observed between the development of metastasis and both clinical fracture [odds ratio (OR), 6.107; 95% confidence interval (CI), 1.08–34.70] and local recurrence (OR, 6.48; 95% CI, 1.03–40.87). Patients presenting with both a clinical fracture and local tumor recurrence require more rigorous clinical observation due to the significantly elevated risk of disease progression.

## Introduction

Giant cell tumor of bone (GCTB) comprises primarily intramedullary bone tumors and accounts for 4–5% of all bone tumors based on a multicenter study including 103 patients between 1980 and 2008 (1). GCTBs are benign lesions; however, several published reports of pulmonary metastasis exist (2). Pulmonary metastasis is detected in ~2–5% of cases of GCTB and is associated with poor treatment outcomes (3). The formation of an ossified rim is a prominent finding in both recurrent and metastatic GCTBs (4). These types of tumors are composed of reactive multinuclear cells expressing receptor activators of nuclear factor-kappa B (RANK) (5), resembling osteoclasts. Previous reports suggest there are overlapping markers for GCTB that are similar to those of osteoclasts, such as tartrate-resistant acid phosphatase (6), cathepsin K (7), carbonic anhydrase II (8) and calcitonin receptor (9).

A population-based study reported that the prevalence of GCTB is 80% in patients aged 20–40 years (10). Although GCTBs occur at multiple sites in the body, the predominant site is the end of a long bone and around the knee, which account for over half of the total number of reported cases (11–14). The clinical manifestations of GCTB comprise swelling, pain and pathological fracture (15). The first-line therapy for GCTB comprises curettage using a high-speed burr to reduce GCTB recurrence (16,17). Patients with GCTB must undergo long-term follow-up as recurrence and metastasis may occur up to 20 years postoperatively (18).

The diagnosis of GCTB comprises clinical observation, radiographs and histopathological analysis (19). The typical clinical manifestations of GCTB are swelling, local pain and pathological fracture (20). Dynamic contrast-enhanced MRI has increased the accuracy of diagnosing GCTBs (21). Additionally, molecular research has provided insights into diagnostic markers, such as p53, p63, kinectin 1, rho-associated, coiled-coil-containing protein kinase 1, nebulin and sterile alpha motif and leucine zipper containing kinase AZK, among others, related to GCTBs (22–24). Pulmonary metastasis is difficult to diagnose in early-stage GCTBs and is more likely to be discovered in recurrent cases (25). The development of pulmonary metastasis from primary lesions may take months to years (26,27).

The aim of the present study was to evaluate patient outcomes and identify any influencing factors in GCTB-induced pulmonary metastasis.

## Materials and methods

### Patient selection

The medical records of 50 patients with GCTB treated in Khon Kaen Hospital (Khon Kaen, Thailand) from January 2016 to December 2021 were retrospectively analyzed. Patients with incomplete medical records were excluded from the study. Ethical approval from The Institute Review Board in Human Research of Khon Kaen Hospital was obtained prior to the initiation of the study (approval no. KEXP65041).

Pulmonary metastasis was confirmed by considering the tumor characteristics, namely single or multiple pulmonary nodules differentiated from developing abnormal lesions, with rounded, well-defined opacities on chest computed tomography (CT). Metastasis was confirmed with subsequent CT if the lesions had increased in number and size.

The collected data comprised the patients' demographic and clinical data, namely the location(s) of the primary lesions, local recurrence history and metastasis. Follow-up durations were defined as the time from the first evaluation for primary treatment to local recurrence and from diagnosis of the primary tumor to pulmonary metastasis. Additional parameters were the treatment type for local recurrence, metastasis treatment, treatment course and follow-up events.

**Figure 1.**
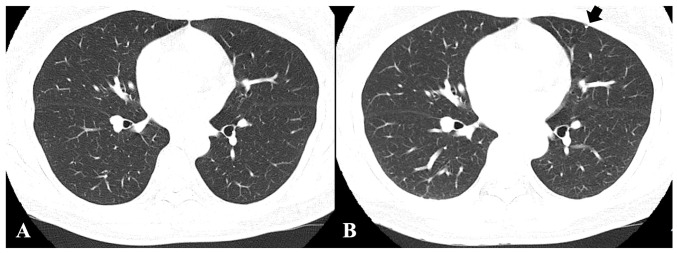
Giant cell tumor of bone in the distal femur without pulmonary metastasis. Chest computed tomography at the (A) first visit and at (B) 12-month follow-up. At follow-up, the patient had a small nodule measuring 4 mm (black arrow) in the left posterior lower lung.

To meet the study purpose, radiographic images, namely CT, X-rays and magnetic resonance images were reviewed. CT images and chest X-rays were evaluated to confirm pulmonary metastasis. Follow-up chest CT images were screened for metastatic nodule development and progression to evaluate treatment efficacy. Follow-up chest CT images were analyzed to measure and evaluate the course of metastatic nodules receiving treatment. The chest CT evaluations determined whether the metastatic nodules were advanced, stationary or reduced (Fig. 1).

### Statistical analysis

Data analysis was conducted using Microsoft Excel version 16.76 (Microsoft Corporation) for editing, sorting and coding. The final Excel file was subsequently imported into SPSS software (version 27; IBM Corp.) for statistical analysis. Continuous variables are expressed as the mean ± standard deviation. The normality of the data was tested using the Kolmogorov-Smirnov test. Unpaired Student's t-test was used to analyze statistical differences between group means. Categorical variables were presented as percentages and the chi-square test was used to compare categorical variables, including patients' clinical characteristics. If the expected value was <5 for >20% of the total cells, Fisher's exact test was used. P<0.05 was considered to indicate a statistically significant difference. Univariate and multivariable logistic regression analyses were conducted to evaluate the risk factors for GCTB-induced pulmonary metastasis. An alternative method, namely the continuity correction, was used for logistic regression analysis on contingency tables containing zero cell counts. The number of events and non-events in studies with zero cell counts was increased by 0.5. Variables with a P<0.10 in the univariate logistic regression analyses and other variables of known clinical relevance were included in the multivariable logistic regression analyses. The regression was performed with 95% confidence intervals (CIs). Univariate logistic regression models with 95% CI were used to conduct univariate [odds ratio (OR)] and multivariable analysis [adjusted odds ratio (AOR)]. An AOR is a statistical measure that has been modified to accommodate the influence of additional predictor variables, including age at diagnosis, axial location of GCTB, fracture and neurological deficit, stage III, and 1 and >1 local recurrences within a model. This measure is particularly valuable in illustrating the impact of a specific predictor variable on the likelihood of an event occurring, while controlling for the influence of other predictor variables. The logistic regression model is prone to being affected by small-sample bias (28). In medical literature, a commonly adopted lower limit for developing prediction models that forecast a binary outcome is an events per variable (EPV) of 10 (29,30). The EPV, representing the smaller count between the number of subjects who experienced the outcome and those who did not, is calculated by dividing it by the number of predictor variables utilized in building the prediction model. In the present study, with 50 events (cases) and six predictor variables including age at diagnosis, location of GCTB, clinical presentation, Campanacci stage, no. of local recurrences and treatment for local recurrence, the resulting EPV is ~8, falling below the recommended threshold of 10. The Firth method (Firth's Bias-Reduced Logistic Regression), named after its creator, utilizes a penalized likelihood approach to mitigate the impact of small-sample bias in maximum likelihood estimation (31). The Firth method was used for small-sample analysis in STATA version 16.0 (StataCorp LP). The variance inflation factor (VIF) was also calculated to confirm multicollinearity. All methods were performed in accordance with the relevant guidelines described by Altman *et al* (32).

## Results

### Patient demographics and clinical data

Of the 50 patients with GCTB, 26 (52.0%) were female and 24 (48.0%) were male, with a female-to-male ratio of 1.08:1.0 (Table I). The primary tumor sites in an extremity were the proximal humerus (n=2), distal radius (n=4), distal humerus (n=0), distal ulna (n=5), distal femur (n=17), proximal femur (n=3), proximal tibia (n=7), proximal fibula (n=3), distal tibia (n=2), talus (n=0), calcaneus (n=1), hand (n=2) and foot (n=1). Additionally, primary tumors were located in the axial locations in the spine (n=0), sacrum (n=2) and pelvis (n=1). Primary GCTBs occurred most often in the distal femur (n=17), accounting for 34% of all patients. Of the 50 patients evaluated in this study, 47 (94%) were primary cases and 3 (6%) were recurrent cases.

### Clinical characteristics

Pain, the presence of a mass, pathological fracture and neurological deficit were significant parameters in identifying and diagnosing the possibility of a tumor (Table I). Pain in 27 patients and pathological fracture in 10 patients accounted for two notable contributors at 54.0 and 20.0% of the cases, respectively. According to the Campanacci grading system (17), Campanacci grade I tumors were reported in 2% of the patients (n=1), Campanacci grade II tumors were reported in 22% of patients (n=11) and Campanacci grade III was most frequent being reported in 76% of patients (n=38). The average period from treatment to recurrence was 13.5±12.5 months. The mean time from primary GCTB to pulmonary metastasis diagnosis was 11.7±9.4 months.

### Mode of treatment

Of the 50 patients with GCTB, 3 patients (6%) received simple curettage without local adjuvant therapy and 35 (70%) received extended curettage with local adjuvant therapy (Table I). Additionally, 11 patients (22%) underwent wide resection, and amputation or disarticulation was necessary for 1 patient (2%).

### Recurrence and metastasis

The average follow-up duration was 26.3 months, during which 74% of the patients (n=37) were without local recurrence (Table I). However, 12 patients (24%) developed recurrence once and 1 patient (2%) developed recurrence more than once.

A total of 41 patients (n=82) did not develop metastasis. From the cohort of 9 patients (18%) who developed pulmonary metastasis, three had single lesions as follows: Right upper lung (n=1; 2%); right middle lung (n=1; 2%); and left upper lung (n=1; 2%). Of the remaining 6 patients, 3 patients (6%) developed multiple lesions in both lungs, 2 patients (4%) developed multiple lesions in the right lung and 1 patient (2%) developed multiple lesions in the left lung.

Of the 9 pulmonary metastasis cases, 5 reported fractures (Table II). Within this group of patients, 2 individuals developed tumor recurrence whereas the remaining 7 patients developed new primary tumors. Of the 2 cases with local recurrence, 1 underwent extended curettage and 1 underwent wide resection. None of the patients died from pulmonary metastasis in the present study.

### Risk factors for pulmonary metastasis

In the present study, pulmonary metastasis occurred in 9 of the 50 patients with GCTB. GCTB occurrence was more frequent in patients aged ≥35 years compared with those aged <35 years in patients with Campanacci grade III tumors (8 vs. 1 patient, respectively; Table II). There was a statistically significant association between GCTB occurrence and age (P=0.045; Table III). The pulmonary metastasis incidence rate among patients with fractures was 55.5% (5/9), which was statistically significant (P=0.024). Similarly, in patients with >1 local tumor recurrence, the pulmonary metastasis incidence rate was 11.11% (1/9) and was statistically significant (P=0.038). Local treatment was demonstrated to be a significant risk factor for developing pulmonary metastasis (P=0.035; Table III). In the present study, sex, location of GCTB, tumor type, Campanacci stage, treatment for GCTB and mean time from treatment to local recurrence were not significantly associated with pulmonary metastasis (P=0.142, P=0.560, P=0.080, P=0.818, P=0.577 and P=0.251, respectively).

Univariate analysis using Firth's Bias-Reduced Logistic Regression revealed a statistically significant association between GCTB-induced pulmonary metastasis and fractures (OR, 7.89; 95% CI, 1.69–36.65, P=0.008; Table IV). Local treatment requiring curettage with wide resection emerged as a significant risk factor for developing pulmonary metastasis (OR, 10.73; 95% CI, 1.61–71.56, P=0.014; Table IV). Multivariable analysis revealed two independent risk factors for developing pulmonary metastasis. Patients who presented with pathological fractures had an increased risk of developing pulmonary metastasis that was 6.107 times (AOR, 6.107; 95% CI, 1.075–34.70) higher than that of patients without pathological fractures (P=0.041). Local recurrence increased the risk of pulmonary metastasis by 6.480 times (AOR, 6.480; 95% CI, 1.027–40.87; P=0.047; Table V). Multicollinearity tests conducted on the model did not detect a significant level of multicollinearity among any of the included covariates with VIF <1.20.

## Discussion

Metastasis of GCTBs is uncommon, as the rate of metastasis varies from 1–9% across previous studies and 3% of GCTBs metastasize to the lung (33–38). In the present study, the prevalence of metastasis was 18%, which differed from that in previously published reports (39–44). In the present study, all nine patients had pulmonary metastases. Among nine pulmonary metastases, recurrent patients showed higher incidences of lung metastasis (66.66%; 2/3) than non-recurrent patients (14.89%; 7/47). Several risk factors are associated with the onset of metastasis, such as local tumor recurrence, a delay in seeking treatment and pathological fractures being the most statistically significant (45,46). Performing tissue biopsies on every patient with a lung mass suspected of being pulmonary metastasis from GCTB is not feasible. It is important to balance the benefits of obtaining a definitive diagnosis with the potential risks, resource allocation and the well-being of the patient. Wang *et al* (47) defined the pulmonary metastasis from GCTB as follows: i) The development of abnormal lesions, either single or multiple pulmonary nodules, rounded and well-defined opacities on chest CT; and ii) there should be evidence of growth during the follow-up period, either in the number or size of lesions.

Pathological fracture is significantly associated with pulmonary metastasis. Previous studies have reported varying incidences (5.3–11%) of pathological fractures among patients diagnosed with pulmonary metastasis (47,48). The rate of pulmonary metastasis from GCTB is relatively high (18%) in the present study due to a delay in patients seeking treatment, leading to pathological fracture. The present study demonstrated that up to 20% of patients presented with pathological fractures and in 55.5% of the cases of pulmonary metastasis fractures were identified as independent risk factors for developing pulmonary metastasis at a rate 6.107 times higher compared with patients who did not experience fractures. The pathological fracture was a critical risk factor for developing metastasis. Faisham *et al* (38) reported that Campanacci grade III is a risk factor for pulmonary metastasis. In the present study, 76% of primary cases had Campanacci stage grade III tumors, which later resulted in metastasis in 7 patients (77.7% of malignant cases).

Local tumor recurrence and metastasis have a positive association (45,49). Similar to findings reported in previous studies, the present study demonstrated that local tumor recurrence significantly increased the risk of pulmonary metastasis. Of the 9 patients with pulmonary metastasis, 5 patients (55.5%) experienced local recurrence at least once and this association was confirmed in both univariate and multivariable analysis. Local curettage and local adjuvant therapy can help prevent local recurrence (50). Wide resection effectively reduces tumor burden in recurrent and pathological fracture cases (51,52).

In addition to local treatment, new drugs have been developed for the treatment of GCTBs. Denosumab was previously introduced for the treatment of advanced and metastatic GCTB and it binds to the RANK ligand-receptor activator (53). Denosumab inhibits the recruitment of osteoclast-like giant cells and prevents osteolysis (53–55). The bisphosphate zoledronic acid, also targets the neoplastic stromal cells in GCTBs (56). In the present study, denosumab was administered to 3 of the patients with pulmonary metastatic cancer and the remaining 6 patients underwent observation only, with no radiation or resection. The present study demonstrated that none of the patients died as a result of pulmonary metastasis from GCTB. Resection of metastatic lesions or administration of denosumab is considered in cases where there is an increase in the size of the metastatic lesion(s) and the metastatic lesion(s) are causing symptoms (57).

Although no mortality was reported in the present study, the reported mortality rate for metastatic GCTB cases ranges from 0–23% (58), which is a major concern. The prognosis is good after timely and appropriate surgical resection for patients with pulmonary metastasis, with a 71–100% survival rate at the last follow-up (59). Denosumab, a monoclonal antibody that inhibits bone breakdown both in normal and tumor-related contexts, by preventing the formation and activation of multinuclear osteoclasts or giant cells mediated by receptor activator of nuclear factor κβ, is being considered as a potential treatment option for pulmonary metastasis in unresectable GCTB (60).

In the present study, most patient cases were managed by close observation. Therefore, pulmonary metastasectomy was not performed immediately post-diagnosis. Metastasectomy is only advisable under conditions where the patient appears to have progressing metastasis. Local tumor recurrence and pathological fracture are independent risk factors for developing pulmonary metastasis from GCTB. Therefore, in cases with local recurrence or pathological fracture, more aggressive treatment, such as wide resection, should be performed to reduce the local recurrence rate and lower the risk of pulmonary metastasis.

The limitation of this study was the small sample size. There were 50 cases of GCTB and only 9 patients developed pulmonary metastasis. Therefore, a larger cohort of patients is required to validate the risk factors associated with GCTBs that were identified in the present study.

There are a number of unanswered questions in regard to the effective treatment of GCTBs, especially regarding the high recurrence rates and adverse effects observed upon systemic therapy (61). There is a need to investigate alternative therapeutic strategies to effectively treat pulmonary metastasis of GCTBs.

To conclude, pulmonary metastasis from GCTB was not uncommon in the present study. CT chest scan should be performed in each patient with GCTB as the rate of pulmonary metastasis from GCTB was relatively high (18%) in the present study. Local recurrence and pathological fracture were associated with developing pulmonary metastasis. It is unnecessary to perform pulmonary metastasectomy immediately. Additionally, a biopsy of metastatic lesions developed from GCT of bone is also unnecessary. Close observation of patients with metastasis is essential and serial imaging is recommended in every case. More studies are required that evaluate the molecular mechanisms of GCTB.

## Figures and Tables

**Table I.** Patient demographic and clinical data.

Patient characteristic	Result
Mean patient age, years (mean ± SD)	36.0±17.2
Follow-up duration, months (mean ± SD)	26.3±18.4
Patient sex, n (%)	
Female	26 (52.0)
Male	24 (48.0)
Duration from first treatment to first appearance of recurrence, (mean ± SD)	13.5±12.5
Primary GCTB diagnosis to first appearance of pulmonary metastasis, (mean ± SD)	11.7±9.4
Primary tumor location, n (%)	
Extremity	
Proximal humerus	2 (4.0)
Distal humerus	0 (0.0)
Distal radius	4 (8.0)
Distal ulna	5 (10.0)
Proximal femur	3 (6.0)
Distal femur	17 (34.0)
Proximal tibia	7 (14.0)
Proximal fibula	3 (6.0)
Distal tibia	2 (4.0)
Talus	0 (0.0)
Calcaneus	1 (2.0)
Hand	2 (4.0)
Foot	1 (2.0)
Axial	
Spine	0 (0.0)
Sacrum	2 (4.0)
Pelvis	1 (2.0)
Tumor type, n (%)	
Primary	47 (94.0)
Recurrence	3 (6.0)
Clinical presentation, n (%)	
Incidental finding	0 (0.0)
Pain	27 (54.0)
Mass	12 (24.0)
Pathological fracture	10 (20.0)
Neurological deficit	1 (2.0)
Campanacci radiographic stage, n (%)	
I	1 (2.0)
II	11 (22.0)
III	38 (76.0)
Mode of treatment for the primary tumor, n (%)	
Simple curettage (without local adjuvant therapy)	3 (6.0)
Extended curettage (with local adjuvant therapy)	35 (70.0)
Wide resection	11 (22.0)
Amputation/disarticulation	1 (2.0)
Local recurrence (number of tumors), n (%)	
0	37 (74.0)
1	12 (24.0)
>1	1 (2.0)
Treatment of local recurrence, n (%)	
None (no local recurrence)	38 (76.0)
Simple curettage (without local adjuvant therapy)	0 (0.0)
Extended curettage (with local adjuvant therapy)	4 (8.0)
Wide resection	3 (6.0)
Amputation/disarticulation	2 (5.0)
Adjuvant therapy	2 (5.0)
Mixed (curettage with adjuvant therapy and wide resection)	1 (2.0)
Location/extension of pulmonary metastasis	
Single lesion	
None	41 (82.0)
Right upper lung	1 (2.0)
Right middle lung	1 (2.0)
Right lower lung	0 (0.0)
Left upper lung	1 (2.0)
Left lower lung	0 (0.0)
Multiple lesions	
Right lung	2 (4.0)
Left lung	1 (2.0)
Both lungs	3 (6.0)
Treatment of pulmonary metastasis, n (%)	
None/no metastasis	41 (82.0)
Observation	6 (12.0)
Bisphosphonate	0 (0.0)
Denosumab	3 (6.0)
Chemotherapy	0 (0.0)
Resection	0 (0.0)
Radiation	0 (0.0)

SD, standard deviation.

**Table II.** Clinical data of patients with pulmonary metastasis.

Patient number	Age, years	Location of GCTB	Clinical presentation	Tumor type	Campanacci radiographic stage	Treatment of GCTB	No. of local recurrences	Treatment of local recurrence	Time from diagnosis of the primary GCTB to diagnosis of pulmonary metastasis, months	Treatment for pulmonary metastasis	Follow-up time, months
1	46	Distal femur	Fracture	Primary	III	Curettage	>1	Wide resection	15	Denosumab	20
2	68	Distal femur	Fracture	Primary	III	Curettage	1	Amputation	0	Observation	6
3	58	Distal femur	No fracture	Primary	III	Wide resection	No recurrence	None	26	Observation	26
4	39	Distal femur	No fracture	Primary	III	Wide resection	No recurrence	None	14	Observation	31
5	42	Distal femur	Fracture	Primary	III	Wide resection	No recurrence	None	2	Observation	6
6	42	Distal femur	Fracture	Primary	III	Curettage	1	Mixed, curettage with adjuvant therapy and wide resection	14	Denosumab	29
7	39	Proximal tibia	No fracture	Recurrent	III	Curettage	1	Extended curettage	3	Denosumab	7
8	65	Proximal femur	Fracture	Primary	II	Curettage	No recurrence	None	7	Observation	8
9	18	Distal femur	No fracture	Recurrent	II	Curettage	1	Wide resection	24	Observation	8

GCTB, giant cell tumor of bone.

**Table III.** Characteristics of the patients with and without pulmonary metastasis.

Factor	Without lung metastasis (N=41)	With lung metastasis (N=9)	P-value
Mean age ± SD, years	33.8±16.8	46.3±15.4	0.045^a^
Sex, n (%)			
Female	19 (73.1)	7 (26.9)	0.142^b^
Male	22 (91.7)	2 (8.3)	
Location of GCTB			
Extremity	38 (82.6)	8 (19.1)	0.560^b^
Axial	3 (75.0)	1 (25.0)	
Clinical presentation			
No fracture	35 (89.7)	4 (10.3)	0.024^b^
Fracture	5 (50.0)	5 (50.0)	
Neurological deficit	1 (100.0)	0 (0.0)	
Tumor type			
Primary	40 (85.1)	7 (14.9)	0.080^b^
Recurrence	1 (33.3)	2 (66.7)	
Campanacci stage			
I	1 (100.0)	0 (0.0)	0.818^b^
II	9 (81.8)	2 (18.2)	
III	31 (81.6)	7 (18.4)	
Treatment for GCTB			
Curettage	32 (84.2)	6 (15.8)	0.577^b^
Wide resection	8 (72.7)	3 (27.3)	
Mixed	1 (100)	0 (0.0)	
No. of local recurrences			
No recurrence	33 (89.2)	4 (10.8)	0.038^b^
1	8 (66.7)	4 (33.3)	
>1	0 (0.0)	1 (100.0)	
Mean time from treatment to local recurrence ± SD, months	16.4±14.7	9.0±7.1	0.251^a^
Treatment for local recurrence			
None	34 (89.5)	4 (10.5)	0.035^b^
Curettage	3 (75.0)	1 (25.0)	
Wide resection	2 (40.0)	3 (60.0)	
Adjuvant therapy	2 (100.0)	0 (0.0)	
Mixed	0 (0.0)	1 (100.0)	

a Unpaired Student's t-test.

b Fisher's exact test. GCTB, giant cell tumor of bone; SD, standard deviation.

**Table IV.** Univariate analysis of the factors associated with pulmonary metastasis from GCTB using the Firth's Bias-Reduced Logistic Regression method.

Factor	Without lung metastasis (n=41)	With lung metastasis (N=9)	Odd ratio	95% CI	P-value
Mean age ± SD	33.8±16.8	46.3±15.4	1.04	0.99–1.08	0.058
Sex					
Female (ref.)	19 (73.1)	7 (26.9)	1	-	-
Male	22 (91.7)	2 (8.3)	0.29	0.06–1.37	0.117
Location of GCTB					
Extremity (ref.)	38 (82.6)	8 (19.1)	1	-	-
Axial	3 (75.0)	1 (25.0)	1.96	0.25–15.11	0.526
Clinical presentation					
No fracture (ref.)	35 (89.7)	4 (10.3)	1	-	-
Fracture	5 (50.0)	5 (50.0)	7.89	1.69–36.65	0.008
Neurological deficit	1 (100.0)	0 (0.0)	2.63^a^	0.09–74.76	0.571
Tumor type					
Primary (ref.)	40 (85.1)	7 (14.9)	1	-	-
Recurrence	1 (33.3)	2 (66.7)	9.00	1.03–78.75	0.050
Campanacci stage					
I (ref.)	1 (100.0)	0 (0.0)	1	-	-
II	9 (81.8)	2 (18.2)	0.79^a^	0.02–25.90	0.894
III	31 (81.6)	7 (18.4)	0.71^a^	0.03–19.33	0.842
Treatment for GCTB					
Curettage (ref.)	32 (84.2)	6 (15.8)	1	-	-
Wide resection	8 (72.7)	3 (27.3)	2.06	0.46–9.25	0.346
Mixed	1 (100)	0 (0.0)	1.66^a^	0.06–45.62	0.762
No. of local recurrences					
No recurrence (ref.)	33 (89.2)	4 (10.8)	1	-	-
1	8 (66.7)	4 (33.3)	3.94	0.87–17.80	0.075
>1	0 (0.0)	1 (100.0)	22.33^a^	0.78–635.58	0.069
Mean time from treatment to local recurrence ± SD, months	16.4±14.7	9.0±7.1	0.96	0.87–1.05	0.373
Treatment for local recurrence					
None (ref.)	34 (89.5)	4 (10.5)	1	-	-
Curettage	3 (75.0)	1 (25.0)	3.28	0.38−28.21	0.278
Wide resection	2 (40.0)	3 (60.0)	10.73	1.61–71.56	0.014
Adjuvant therapy	2 (100.0)	0 (0.0)	1.53^a^	0.06–37.29	0.793
Mixed	0 (0.0)	1 (100.0)	23.00^a^	0.81–654.23	0.066

P-values were calculated using the Firth method.

a Continuity correction applied to the odd ratio calculation on contingency tables with zero cell counts. SD, standard deviation; ref., reference category for statistical comparison.

**Table V.** Multivariable analysis of the factors associated with pulmonary metastasis from GCTB using the Firth's Bias-Reduced Logistic Regression method.

Factor	P-value	Adjusted odds ratio	95% CI
Age at diagnosis	0.144	1.033	0.988–1.079
Location of GCTB (axial)	0.488	0.355	0.019–6.623
Clinical presentation (fracture and neurological deficit)^a^	0.041	6.107	1.075–34.70
Campanacci stage (III)	0.547	0.562	0.086–3.671
No. of local recurrences (1 and >1)^a^	0.047	6.480	1.027–40.87

Variables with

a P-value <0.10 in the univariate logistic regression analyses and other variables of known clinical relevance were included as basis categories in the multivariable logistic regression analyses, which comprised age at diagnosis, axial location of GCTB, fracture and neurological deficit, stage III, and 1 and >1 local recurrences. GCTB, giant cell tumor of bone.

## Data Availability

The datasets used and/or analyzed during the current study are available from the corresponding author on reasonable request.
